# Mortality From Myocardial Infarction After the Death of a Sibling: A Nationwide Follow‐up Study From Sweden

**DOI:** 10.1161/JAHA.112.000046

**Published:** 2013-04-24

**Authors:** Mikael Rostila, Jan Saarela, Ichiro Kawachi

**Affiliations:** 1 Centre for Health Equity Studies, Stockholm University/Karolinska Institutet, Stockholm, Sweden (M.R.); 2 Åbo Akademi University and University of Helsinki, Vasa, Finland (J.S.); 3 Harvard School of Public Health, Boston, MA (I.K.)

**Keywords:** bereavement, epidemiology, grief, myocardial infarction, Sweden

## Abstract

**Background:**

Death of a sibling represents a stressful life event and could be a potential trigger of myocardial infarction (MI). We studied the association between loss of an adult sibling and mortality from MI up to 18 years after bereavement.

**Methods and Results:**

We conducted a follow‐up study for Swedes aged 40 to 69 years between 1981 and 2002, based on register data covering the total population (N=1 617 010). Sibling deaths could be observed from 1981 and on. An increased mortality rate from MI was found among women (1.25 CI 1.02 to 1.54) and men (1.15 CI 1.03 to 1.28) who had experienced death of an adult sibling. An elevated rate some years after bereavement was found among both women (during the fourth to sixth half‐years after the death) and men (during the second to sixth half‐years after the death), whereas limited support for a short‐term elevation in the rate was found (during the first few months since bereavement). External causes of sibling death were associated with increased MI mortality among women (1.54 CI 1.07 to 2.22), whereas nonexternal causes showed associations in men (1.23 CI 1.09 to 1.38). However, further analyses showed that if the sibling also died from MI, associations were primarily found among both women (1.62 CI 1.00 to 2.61) and men (1.98 CI 1.59 to 2.48).

**Conclusions:**

Our study provided the first large‐scale evidence for mortality from MI associated with the death of a sibling at an adult age. The fact that findings suggested associations primarily between concordant causes of death (both died of MI) could indicate genetic resemblance or shared risk factors during childhood. Future studies on bereavement should carefully deal with the possibility of residual confounding.

## Introduction

It is well known that the risk of myocardial infarction (MI) is influenced by stress and stressful life events.^1–2^ Bereavement represents a stressful life event, and studies suggest that the risk of acute MI increases after the loss of a significant person.^3^ For instance, cardiovascular disease accounts for 20% to 53% of the excess deaths during the early weeks and months of spousal bereavement.^4–8^ Another study showed an increased risk of MI in bereaved parents after the loss of a child.^9^ However, the contribution of MI to excess deaths after other types of familial losses and in a longer‐term perspective is unclear.

The least‐studied familial relationship in the bereavement literature is that of adult siblings.^10–12^ The impact of grief after the loss of an adult sibling has been largely overlooked. To the extent that siblings are also beloved and provide companionship and support, one would expect that the death of an adult sibling—as much as the death of other family members (eg, spouse, parents, children)—could be considered a stressful life event and a potential trigger of MI. In fact, the death of a sibling often represents the loss of the longest and most intimate relationship of a person's lifetime.^13^ Some studies even suggest that the death of a sibling is more disruptive and involves a more severe grief process than other familial deaths.^12,14^

Bereavement could lead to both acute and chronic mental stress and thereby influence the risk of MI.^15^ Bereavement could trigger MI through acute psychophysiological stress mechanisms, which have been observed to follow episodes of intense psychogenic shock and are also known as “takotsubo cardiomyopathy,” “transient left ventricular apical ballooning,” or “broken heart syndrome.”^16–19^ Deaths from a broken heart usually occur within the first few hours and days after a stressful event.^16,19^ Chronic stressors after bereavement could, however, also lead to pathophysiological changes in the sympathetic nervous system, the hypothalamic‐pituitary‐adrenal axis, and the immune system.^9,20^ It has been suggested that such mechanisms might work through sympathetic nervous system–induced inflammatory mechanisms and thereby contribute to disease risk subsequent to bereavement.^21–22^ Pathophysiological changes could lead to MI months or years after bereavement; an increased risk could occur if the sibling death is unexpected and hence more stressful.^14^ In addition, deleterious coping responses, such as smoking, increased alcohol consumption, and poor diet and exercise habits, could follow bereavement.^23–24^ Such behaviors are likely to contribute to increased risk of MI over the longer term. Although there are strong reasons to believe that a sibling death and the stress and grief that are involved could have an impact on surviving siblings through the aforementioned mechanisms, it should be emphasized that knowledge regarding active mechanisms is scarce.

Previous studies have suggested sex differences in the association between bereavement and health. Men, in particular, appear more vulnerable during the earlier months of bereavement, whereas the risk period appears to be more prolonged for women,^25^ which might be due to the influence of persistent grief. Middle‐aged women are especially vulnerable to acute stress levels after grief (ie, the broken heart syndrome).^18^ Consequently, there could be significant sex differences in the pattern of association between the loss of a sibling and the risk of MI in the surviving relative.^14^

Siblings share a similar biological predisposition to death and disease, which makes confounding by genetic inheritance likely. Siblings also share many environmental exposures during childhood and adolescence (eg, parental education, unhealthy eating habits, etc). An important threat to causal inference therefore is the possibility that the death from MI in the same sibling group is related to shared biological and genetic similarities (ie, there is confounding of the relationship by an unobserved third variable, in this instance, shared biologic predisposition).^14^ If a sibling dies of a heart attack and the other sibling dies later, this may be a marker of genetic or biological similarity. One method of getting closer to causal inference is to examine deaths due to specific causes. By studying whether pairs of siblings died of the same specific cause (eg, both died of a heart attack) or a discordant cause may assist in teasing out causation from confounding.

Our aim was to conduct a large‐scale longitudinal study on mortality from MI after the loss of an adult sibling, using intergenerational linked data from nationwide Swedish registers. We postulated that the association between a sibling's death and mortality from MI will depend on the time interval since the sibling's death, the sex of the remaining sibling, and the cause of death.

## Methods

The data, approved by the Regional Ethical Review Board of Karolinska Institutet in November 11, 2002 (decision no. 02‐481), come from the Swedish Work and Mortality Data. The Swedish Work and Mortality Data provide multiple‐linked data of national Swedish routine registers, maintained at the Centre for Health Equity Studies in Stockholm.

In the study, all persons born in Sweden during the period 1932–1962 and alive at the end of 1980 were linked to their mother, provided that she was born in Sweden and alive at the same time. Hence, sibling groups are identified through their mother. Singletons were excluded from analysis. We restrict the data to people aged ≥40 years, because very few persons die from MI in young adulthood. Therefore, the study included persons aged 40 to 69 years, who could be observed during 1981–2002.

We included individual‐level information about basic sociodemographic variables (age, socioeconomic status, marital status, number of children, number of siblings, region of residence, and calendar year) to proxy regional and social differences in mortality from MI, as well as the month and specific cause of death for all persons who died during the study period. Socioeconomic status distinguished blue‐collar workers, white‐collar workers, self‐employed persons, and people outside the labor market. Marital status consisted of the categories of married, previously married, and never married. Number of children and number of siblings were treated as categorical variables. Region of residence refers to each person's county of residence and consisted of 26 different categories. All covariates except age and calendar year were measured at the end of 1980, which antedated any sibling death. We distinguished deaths from MI, which have *International Classification of Diseases, Revision 8* (ICD‐8) codes 410 and 795, ICD‐9 codes 410 and 798, and ICD‐10 codes I21 and I46. Sibling deaths from MI were contrasted to deaths from other cardiovascular disease (ICD‐8 and ICD‐9 codes 411 to 438 and ICD‐10 codes I22 to I52 and I60 to I69), cancer (ICD‐8 and ICD‐9 codes 140 to 239 and ICD‐10 codes C00 to D48), suicide (ICD‐8 and ICD‐9 codes E950 to E959 and ICD‐10 codes X60 to X84), and other external causes (ICD‐8 codes E807 to E949 and E960 to E999, ICD‐9 codes E800 to E949 and E960 to E999, and ICD‐10 codes V01 to X59 and X85 to Y98). “Other nonexternal cause” refers to all other codes.

All people who experienced a sibling's death at some time during the observation period were included, whereas those who did not experience a sibling's death composed a 10% random sample. In the statistical analyses, people from each group were weighted according to their sampling proportion using normalized weights to correct for inflated *t* statistics. The death of sibling is a time‐varying feature, which means that when a sibling died, the surviving sibling changed status from being a nonbereaved to being a bereaved person. We estimated standardized mortality rates from MI using Poisson regressions and focused on the rate ratio of bereaved and nonbereaved persons. Separate analyses were conducted for men and women. Covariates included in the regressions were age, calendar year, region of residence, socioeconomic status, marital status, number of children, and number of siblings. When stepwise adding each of these in the aforementioned order, all except number of siblings in women improved the statistical fit of the models. Throughout the report, the level of statistical significance referred to is 0.05.

## Results

In total, 65 802 men and 65 118 women experienced a sibling's death, and 469 and 134 of them subsequently died from MI (Table 1). Corresponding numbers in the nonbereaved group were 5172 MI deaths among 750 390 men and 1225 MI deaths among 65 118 women. The unstandardized mortality rate of bereaved persons was roughly twice that of nonbereaved persons (in Table 2: 0.85/0.45 for men and 0.24/0.11 for women).

**Table 1.** Some Descriptive Statistics by Sex of the Index Persons

	Men	Women
No. of ever bereaved persons	65 802	65 118
No. of nonbereaved persons	750 390	735 700
No. of deaths from myocardial infarction		
In bereaved persons	469	134
In nonbereaved persons	5172	1225
No. of person‐years in		
Bereaved persons	552 886	548 381
Nonbereaved persons	11 383 335	11 105 989
Percent of all sibling deaths from		
Myocardial infarction	10.4	10.4
Other cardiovascular disease	13.3	13.3
Cancer	39.8	39.7
Other nonexternal cause	20.9	21.0
Suicide	6.8	7.0
Other external cause	8.6	8.6

Number of nonbereaved persons represents people in the 10% sample of people who did not experience the death of sibling.

**Table 2.** Characteristics of Bereaved and Nonbereaved Persons by Sex

	Men						Women					
	Bereaved			Nonbereaved			Bereaved			Nonbereaved		
	%	No. of Deaths	Rate	%	No. of Deaths	Rate	%	No. of Deaths	Rate	%	No. of Deaths	Rate
Age, y												
40 to 44	16.6	10	0.11	31.5	410	0.11	16.2	3	0.03	31.1	108	0.03
45 to 49	22.4	46	0.37	28.8	833	0.25	21.9	6	0.05	28.6	186	0.06
50 to 54	25.0	89	0.64	21.1	1330	0.55	24.8	24	0.18	21.3	251	0.11
55 to 59	20.7	132	1.15	12.1	1310	0.95	20.9	34	0.30	12.4	315	0.23
60 to 64	11.9	131	1.98	5.1	940	1.61	12.4	43	0.63	5.4	262	0.44
65 to 69	3.4	61	3.24	1.2	349	2.62	3.8	24	1.17	1.3	103	0.72
Socioeconomic status												
Blue‐collar worker	48.0	227	0.85	40.8	2385	0.51	36.0	56	0.28	29.7	451	0.14
White‐collar worker	31.2	123	0.71	40.2	1502	0.33	32.0	29	0.17	39.8	302	0.07
Self‐employed	10.6	54	0.92	10.8	585	0.48	4.3	4	0.17	4.3	49	0.10
Outside labor market	10.2	65	1.16	8.2	700	0.75	27.8	45	0.30	26.1	423	0.15
Marital status												
Married	57.4	262	0.83	60.8	2952	0.43	65.8	72	0.20	68.3	742	0.10
Previously married	8.4	76	1.64	7.5	725	0.85	12.0	35	0.53	10.6	233	0.20
Never married	34.3	131	0.69	31.7	1495	0.41	22.2	27	0.22	21.2	250	0.11
No. of children												
0	28.9	126	0.79	29.6	1389	0.41	17.3	24	0.25	18.9	451	0.21
1	19.2	91	0.86	18.8	923	0.43	19.2	28	0.27	19.0	302	0.14
2	33.3	144	0.78	34.5	1626	0.41	38.8	38	0.18	40.4	49	0.01
>2	18.6	108	1.05	17.1	1234	0.63	24.6	44	0.33	21.7	423	0.18
No. of siblings												
1	17.7	69	0.71	41.3	2085	0.44	18.0	20	0.20	41.7	742	0.16
2	25.6	119	0.84	29.3	1414	0.42	25.1	33	0.24	29.0	233	0.07
>2	56.8	281	0.90	29.4	1673	0.50	56.8	81	0.26	29.3	250	0.08
Total	100.0	469	0.85	100.0	5172	0.45	100.0	134	0.24	100.0	1225	0.11

Deaths refer to deaths from myocardial infarction.

Descriptive statistics for region of residence and calendar year are not displayed.

Number of person‐years (total risk time) is 552 886 in bereaved men, 11 383 335 in nonbereaved men, 548 381 in bereaved women, and 11 105 989 in nonbereaved women.

% refers to percentage of total risk time.

Rate is number of deaths per person‐years multiplied by 1000.

Bereaved persons were slightly older than nonbereaved persons, somewhat more of them had a lower socioeconomic position, and they had more siblings, which is expected considering that the likelihood of observing a sibling's death must be higher in larger sibling groups (Table 2). We accounted for distributional differences between bereaved and nonbereaved persons by using the control variables, which generally provided good statistical fit. Hence, throughout the analyses we estimated standardized mortality rate ratios (ie, the ratio of the death rate of persons who experienced the death of a sibling and the death rate of persons who did not experience the death of a sibling).

The standardized rate of fatal MI was also notably higher in bereaved persons than in nonbereaved persons (Table 3). Among women, the mortality rate ratio of bereaved to nonbereaved persons was 1.25 (95% CI 1.02 to 1.28), whereas in men it was 1.15 (95% CI 1.03 to 1.28). In most subcategories of the control variables, there was an association between bereavement and mortality from MI, but the statistical power was generally too small to facilitate any detailed conclusions on this point (Table 4).

**Table 3.** Effect of Sibling's Death on Mortality From Myocardial Infarction, by Cause of Sibling's Death

	Men		Women	
Cause of sibling's death				
All causes	1.15	(1.03 to 1.28)	1.25	(1.02 to 1.54)
External	0.84	(0.66 to 1.07)	1.54	(1.07 to 2.22)
Not external	1.23	(1.09 to 1.38)	1.18	(0.94 to 1.48)
Suicide	1.13	(0.81 to 1.44)	1.15	(0.55 to 1.75)
Other external	0.62	(0.25 to 0.99)	1.86	(1.41 to 2.30)
Myocardial infarction	1.98	(1.59 to 2.48)	1.62	(1.00 to 2.61)
Not myocardial infarction	1.05	(0.94 to 1.18)	1.21	(0.97 to 1.50)
Other cardiovascular	1.74	(1.40 to 2.16)	1.50	(0.95 to 2.37)
Cancer	0.95	(0.80 to 1.14)	0.86	(0.60 to 1.23)
Other nonexternal	1.03	(0.83 to 1.29)	1.35	(0.92 to 1.96)

Data are standardized mortality ratios (with 95% CIs) between bereaved and nonbereaved persons (ie, the ratio of the death rate of persons who experienced the death of a sibling and the death rate of persons who did not experience the death of a sibling), adjusted for effects of all the control variables.

Control variables included in the estimations are age, calendar year, socioeconomic status, marital status, number of children, number of siblings, and region of residence.

Separate models have been estimated for men and for women.

**Table 4.** Effect of Sibling's Death (From Any Cause) on Mortality From Myocardial Infarction Stratified by Age Category, Socioeconomic Status, Marital Status, Number of Children, and Number of Siblings

	Men		Women	
Age, y				
40 to 44	0.82	(0.44 to 1.54)	0.85	(0.27 to 2.68)
45 to 49	1.31	(0.97 to 1.77)	0.71	(0.31 to 1.62)
50 to 54	1.08	(0.87 to 1.35)	1.47	(0.95 to 2.26)
55 to 59	1.17	(0.97 to 1.41)	1.19	(0.82 to 1.72)
60 to 64	1.17	(0.97 to 1.41)	1.30	(0.92 to 1.82)
65 to 69	1.15	(0.87 to 1.51)	1.44	(0.91 to 2.28)
Socioeconomic status				
Blue‐collar worker	1.08	(0.94 to 1.25)	1.23	(0.92 to 1.66)
White‐collar worker	1.37	(1.13 to 1.66)	1.43	(0.96 to 2.12)
Self‐employed	1.29	(0.97 to 1.72)	1.00	(0.36 to 2.77)
Outside labor market	0.95	(0.74 to 1.24)	1.20	(0.87 to 1.66)
Marital status				
Married	1.19	(1.04 to 1.36)	1.14	(0.87 to 1.48)
Previously married	1.15	(0.91 to 1.47)	1.49	(1.02 to 2.15)
Never married	1.07	(0.89 to 1.29)	1.34	(0.89 to 2.01)
No. of children				
0	1.21	(1.00 to 1.47)	1.17	(0.76 to 1.80)
1	1.24	(1.00 to 1.55)	1.25	(0.83 to 1.87)
2	1.17	(0.98 to 1.40)	1.13	(0.80 to 1.60)
>2	0.99	(0.81 to 1.22)	1.45	(1.04 to 2.03)
No. of siblings				
1	0.99	(0.77 to 1.26)	1.20	(0.76 to 1.90)
2	1.25	(1.03 to 1.51)	1.32	(0.91 to 1.92)
>2	1.16	(1.01 to 1.33)	1.23	(0.95 to 1.60)

Data are standardized mortality ratios (with 95% CIs) between exposed and unexposed index persons (ie, the ratio of the death rate of persons with a deceased sibling and the death rate of persons with no deceased sibling), adjusted for effects of all control variables.

Control variables included in the estimations are age, calendar year, socioeconomic status, marital status, number of children, number of siblings, and region of residence.

The results are based on 5 different specifications for each sex, where in each model with all main effects we have included also the joint effect of sibling's death and the control variable of interest.

**Figure 1.**
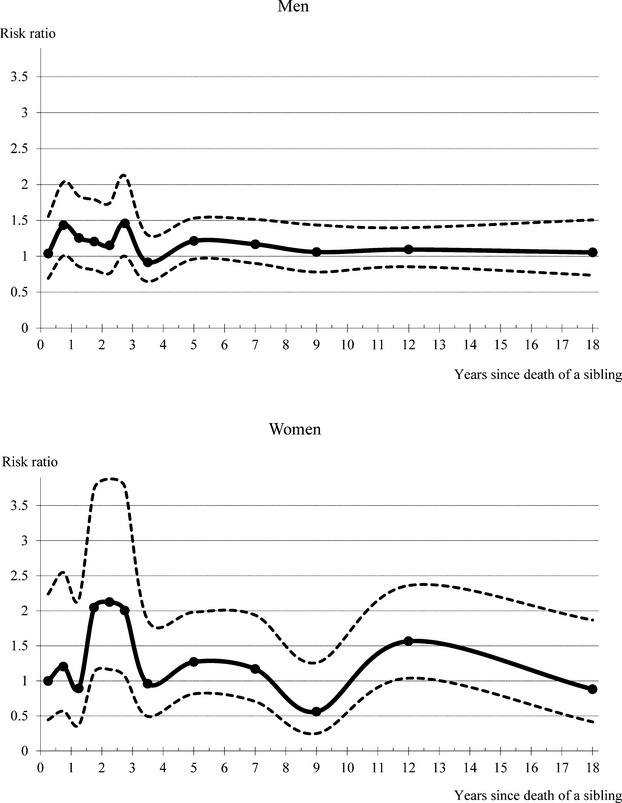
Standardized mortality from myocardial infarction after the death of a sibling compared with nonbereaved persons (with 95% CIs).

We observed an association between a sibling's death and mortality from MI after some years of bereavement (Figure 1). For men, the mortality rate during years 1 to 3 after a sibling's death was ≈30% higher than for nonbereaved men, albeit the elevation was not consistently statistically significant. For women, the mortality rate during years 2 to 3 after a sibling's death was notably higher, or approximately twice that of nonbereaved persons. Longer‐term effects could not be discerned, since there was no pronounced mortality increase ≥3 years after a sibling's death. The sex‐specific patterns were fairly similar in different age groups, albeit the CIs were wide due to relatively few numbers of deaths (Table 5). The percentage of deaths from MI was ≈13% of the total number of deaths during the first 10 years since sibling loss in men and ≈5.5% in women (not shown in table). In general, MI constituted a slightly larger share of bereavement‐related deaths during the first few years of bereavement.

**Table 5.** Mortality From Myocardial According to Time Since the Death of a Sibling by Sex and Age Group

Quarter Since Sibling's Death	Men						Women					
	40 to 69 y		40 to 54 y		55 to 69 y		40 to 69 y		40 to 54 y		55 to 69 y	
Q1 to Q2	1.04	(0.69 to 1.55)	0.51	(0.19 to 1.38)	1.29	(0.83 to 2.02)	1.00	(0.44 to 2.24)	0.62	(0.09 to 4.47)	1.11	(0.46 to 2.70)
Q3 to Q4	1.43	(1.01 to 2.04)	1.47	(0.81 to 2.68)	1.40	(0.91 to 2.16)	1.20	(0.57 to 2.55)	Not applicable		1.60	(0.75 to 3.40)
Q5 to Q6	1.26	(0.86 to 1.84)	1.41	(0.75 to 2.63)	1.17	(0.72 to 1.90)	0.89	(0.37 to 2.16)	2.06	(0.65 to 6.46)	0.47	(0.12 to 1.90)
Q7 to Q8	1.20	(0.81 to 1.79)	1.03	(0.49 to 2.17)	1.28	(0.80 to 2.04)	2.05	(1.12 to 3.74)	2.15	(0.68 to 6.77)	1.96	(0.96 to 3.99)
Q9 to Q10	1.15	(0.76 to 1.74)	1.23	(0.61 to 2.48)	1.10	(0.66 to 1.84)	2.12	(1.16 to 3.88)	0.75	(0.10 to 5.38)	2.54	(1.34 to 4.80)
Q11 to Q12	1.46	(1.00 to 2.12)	0.97	(0.43 to 2.17)	1.67	(1.09 to 2.56)	2.00	(1.07 to 3.76)	2.36	(0.75 to 7.42)	1.84	(0.86 to 3.91)
Q13 to Q16	0.92	(0.65 to 1.30)	1.39	(0.84 to 2.28)	0.69	(0.42 to 1.11)	0.96	(0.49 to 1.86)	1.69	(0.63 to 4.59)	0.69	(0.29 to 1.69)
Q17 to Q24	1.21	(0.96 to 1.53)	1.11	(0.73 to 1.70)	1.25	(0.94 to 1.65)	1.27	(0.81 to 1.98)	1.23	(0.50 to 3.02)	1.25	(0.75 to 2.09)
Q25 to Q32	1.17	(0.90 to 1.51)	1.25	(0.80 to 1.95)	1.12	(0.81 to 1.54)	1.17	(0.71 to 1.94)	1.52	(0.62 to 3.72)	1.03	(0.56 to 1.90)
Q33 to Q40	1.06	(0.78 to 1.44)	1.12	(0.66 to 1.90)	1.02	(0.70 to 1.49)	0.56	(0.25 to 1.26)	0.38	(0.05 to 2.74)	0.60	(0.25 to 1.47)
Q41 to Q60	1.09	(0.85 to 1.40)	0.83	(0.50 to 1.36)	1.21	(0.91 to 1.60)	1.57	(1.04 to 2.36)	1.24	(0.51 to 3.04)	1.63	(1.03 to 2.60)
Q61 to Q88	1.05	(0.74 to 1.51)	1.51	(0.83 to 2.74)	0.90	(0.58 to 1.41)	0.88	(0.41 to 1.87)	1.22	(0.30 to 4.95)	0.77	(0.31 to 1.87)

Data are standardized mortality ratios (with 95% CIls) between exposed and unexposed index persons (ie, the ratio of the death rate of persons with a deceased sibling and the death rate of persons with no deceased sibling), adjusted for effects of all control variables.

Control variables included in the estimations are age, calendar year, socioeconomic status, marital status, number of children, number of siblings, and region of residence.

Models have been estimated separately by sex and age group.

Reference group is nonbereaved persons.

The presented period categorization of time since a sibling's death was the one that fitted the data best. The presented period categorization of time since a sibling's death was the one that fitted the data best.

Estimates for the age group 40 to 69 years correspond to Figure 1, where the dots in that figure have been set at the midpoint of each interval.

More detailed analyses of the cause of the siblings' death revealed that at least part of the association between sibling loss and fatal MI might be due to residual confounding, because it was found primarily in cases where both persons in a sibling pair died from MI (Table 3). In men, the standardized rate ratio for this association was 1.98 (95% CI 1.59 to 2.48), whereas in women it was 1.62 (95% CI 1.00 to 2.61). In contrast, there was no increase in the rate of male MI if the sibling died from an external cause or from any other nonexternal cause than MI. For women, on the other hand, the mortality rate of MI was 54% higher if the sibling died from an external cause (95% CI 1.07 to 2.22) and 86% higher if that cause was not suicide (95% CI 1.41 to 2.30), whereas the rate ratio was lower and nonsignificant if the sibling died from any other cause than MI. A sibling's death from any other cardiovascular disease than MI was also strongly associated with mortality from MI in the index persons. The standardized rate ratio was 1.74 in men (95% CI 1.40 to 2.16) and 1.50 in women (95% CI 0.95 to 2.37). These findings indicate that, at least for men, the association between the loss of a sibling and fatal MI in the surviving relative might be confounded by shared predisposition.

## Discussion

This large‐scale follow‐up study based on the Swedish population register examined bereavement after the death of a sibling as a trigger of MI in the surviving relative. Some initial analyses showed that the death of a sibling was associated with an increased mortality from MI among both men and women. Sibling deaths from external causes (primarily suicides and accidents) were also associated with MI among women. We primarily observed associations with fatal MI some years after a sibling's death but no associations immediately after the death. Analyses of cause‐specific mortality suggested that the associations may not be due to stress‐related mechanisms in men. Hence, we cannot exclude that bereavement effects may reflect the fact that male family members share a similar biological predisposition to death and disease and that they share many environmental exposures.

Our results suggest that if there is an association between the loss of a sibling and MI, it can primarily be discerned some years after bereavement. It could be that adverse coping responses (eg, unhealthy life styles) underlie the association. Hence, deleterious coping responses, such as smoking, increased alcohol consumption, and poor diet and exercise habits, could follow bereavement ^23–24^ and contribute to increased risk of MI. Moreover, chronic mental stress after the death of a sibling could also lead to pathophysiological changes in the hypothalamic‐pituitary‐adrenal axis and the immune system, with health consequences some years after the loss of a sibling.^9,20^ Sympathetic nervous system–induced inflammatory mechanisms could also be a possible contributor to disease risk subsequent to bereavement. Accordingly, one study found elevated levels of the inflammatory markers interleukin‐1 receptor antagonist and interleukin‐6 among bereaved individuals.^21^ However, we found limited support for the fact that sibling loss influences MI through acute psychophysiological stress mechanisms observed to follow episodes of intense psychogenic shock, also known as the broken heart syndrome.^3,16–19^ That no shorter‐term association was found could indicate that adult siblings normally live separate lives and have their own families and therefore it is possible that their primary network (spouse and children) can help them cope with grief and increased stress levels in the immediate aftermath of a sibling's death and therefore postpone the appearance of the association for some years. The results could also reflect that bereavement after the death of a sibling leads to a milder bereavement process. Consequently, other types of losses such as child or spousal deaths could represent more stressful events. Nevertheless, the study of short‐ and long‐term effects of bereavement on MI could still be considered a very crude and indirect test of active mechanisms. More detailed information on active mechanisms linking bereavement and MI is needed to evaluate factors that explain the associations.

Women were found to be more vulnerable to the death of a sibling from all causes compared with men. They also showed an excess mortality rate from MI after sibling deaths from external causes. This finding might reflect the fact that women place more emphasis on social relationships than do men, particularly regarding parents and the family.^26^ The loss of a sibling due to an external and unexpected cause could be especially severe and might have stronger emotional consequences for women, which, in turn, could account for higher stress levels and higher mortality. Accordingly, our previous findings indicate that women's health is more influenced by bereavement than is men's health.^14,27–28^

In general, we found an increased risk of dying from concordant causes among pairs of siblings (ie, in cases where both siblings died of MI). No significant associations were found when siblings died of discordant causes, with the exception of an association among women who experienced the death of a sibling from external causes other than suicide. This finding indicates confounding; that is, that the association might be explained by an unobserved third variable (eg, genetic similarities between siblings or shared childhood environment and family effects). Given the strong genetic similarities between siblings, there could be a higher risk of confounding compared with research on other types of bereavement. Accordingly, studies on twins and genomewide association studies indicate a genetic component of cardiovascular disease.^29–31^ On the other hand, it could be that many deaths from the same cause (ie, both siblings died of MI) still reflect effects of bereavement. MI is highly responsive to bereavement, and previous studies show that cardiovascular disease accounts for a great share of the excess deaths during the early weeks and months of bereavement.^4–8^ Even though siblings died of the same cause, we cannot exclude the possibility that the association, to some extent, could reflect bereavement rather than confounding (ie, one sibling dies of MI and the remaining sibling dies of heart attack due to bereavement rather than genetic vulnerability or shared environmental exposures). Consequently, our analyses may not be completely explained by reversed causality.

Despite the obvious strengths of this study, such as the use of total population register data, large sample size, longitudinal follow‐up, reliable information on mortality from MI, and other included variables, some limitations should be noted. More detailed individual information is required to uncover the actual causal mechanisms that link sibling deaths and MI. Such information could also minimize the possibility of omitted variable bias. Furthermore, information regarding comorbid conditions at baseline or follow‐up and presence and severity of bereavement, menopausal status, psychiatric screening, and other variables is not available in the registers. Such variables could contribute to the understanding of underlying factors. Ideally, one would like to have access to biological and genetic data, detailed information on diseases from medical records, more information on shared childhood social environment and family characteristics, and detailed data on personal and relational characteristics, which, unfortunately, are not included in the registers. On the other hand, our results likely underestimate the true bereavement effect on the risk of MI, because we could study only deaths from MI. For instance, advancements in prevention and medical techniques prevent many fatal heart attacks in today's health care system. Examining incident MIs would provide more precision and greater statistical power for the estimates. The absence of a relationship between sibling loss and deaths from MI during the first few months of bereavement might be explained, for instance, by the fact that nonfatal MI events were not analyzed, and mortality from MI does not necessarily coincide with the specific event of MI but with a time lag. Accordingly, the definition of MI based on ICD codes is confined to transmural MI and is not inclusive of non‐ST‐segment or out‐of‐hospital MI. The study of fatal MI also leaves the possibility that study subjects are exposed to a nonfatal MI after bereavement although they later on die of another cause. This may cause an underestimation of the association with MI. Furthermore, our analyses of deaths from concordant causes as an indication of confounding could also underestimate the “true” effect of bereavement. Many deaths from the same cause (ie, both died of MI) could in fact be related to bereavement processes. Finally, the fact that some of our findings indicate no causal effect by bereavement, which deviates from many other studies, suggests that this research area might suffer from the “file drawer effect” (ie, nonreporting in the literature of nonsignificant results).^32^

From a policy perspective, our findings suggest that the health care system should be concerned about broader “collateral health effects” of illness and death on members of the decedent's social networks.^33^ Our findings also conform to the view that it is important for health care workers to follow bereaved siblings after the death of a sibling and recognize signs of short‐ or long‐term psychosocial stress mechanisms that could lead to a risk of MI.^34^ Triggered cardiovascular events might be prevented with traditional cardiovascular medications (eg, aspirin, β‐blockers, statins) and with stress management.

In summary, our study provided the first large‐scale evidence for mortality from MI associated with the death of a sibling at an adult age. Our findings emphasize that future studies on bereavement and mortality should carefully deal with the possibility of residual confounding by shared biologic and family characteristics. The mechanisms linking the death of a sibling and MI among bereaved persons also need further investigation.
